# Differential Expression of Urinary Exosomal Small RNAs in Idiopathic Membranous Nephropathy

**DOI:** 10.1155/2020/3170927

**Published:** 2020-12-29

**Authors:** Jinshi Zhang, Yifan Zhu, Ruyi Cai, Juan Jin, Qiang He

**Affiliations:** ^1^School of Medicine, Hangzhou Normal University, Zhejiang 310018, China; ^2^Department of Nephrology, Zhejiang Provincial People's Hospital, Zhejiang 310014, China; ^3^People's Hospital of Hangzhou Medical College, Zhejiang 310014, China; ^4^Chinese Medical Nephrology Key Laboratory of Zhejiang Province, Hangzhou, Zhejiang 310014, China

## Abstract

**Background:**

Idiopathic membranous nephropathy (IMN) is a major cause of adult nephrotic syndromes, and reliable noninvasive biomarkers for diagnosis and monitoring are urgently needed. In this study, we performed small RNA (sRNA) sequencing to explore sRNA profiles of urinary exosomes derived from IMN patients and healthy controls (CON) to provide clues for identifying novel noninvasive sRNA biomarkers for IMN.

**Methods:**

Urine samples were collected from five healthy controls and six patients with IMN. High-throughput sequencing was used to screen sRNA expression profiles of urinary exosomes from patients with IMN in two independent cohorts.

**Results:**

Urinary exosomes were successfully isolated and used to obtain exosomal sRNAs. We screened 131 differentially expressed miRNAs, including 28 specifically expressed miRNAs, then explored the top 10 specifically expressed miRNAs in all IMN individuals. The specifically expressed miRNAs and differentially expressed miRNAs provide potential biomarkers for IMN. Additionally, we discovered numerous sRNAs derived from genomic repetitive sequences, which could represent an exciting new area of research.

**Conclusion:**

Herein, we revealed significant differences in expression profiles of urinary exosomal miRNAs and repetitive region-derived sRNAs between patients with IMN and healthy controls. The findings could facilitate the development of potential molecular targets for membranous nephropathy.

## 1. Introduction

Membranous nephropathy (MN) comprises an important etiological factor of adult nephrotic syndrome [1]. A recent large multicentre retrospective study that included 71,151 renal puncture cases in China revealed that the percentage of MN was 23.4%, lower compared to IgA nephropathy but rapidly increasing [2]. MN is categorized into two classes: idiopathic membranous nephropathy (IMN) and secondary membranous nephropathy. The majority of cases are IMN, which is considered an organ-distinct autoimmune disease, and about a third of cases are secondary to familiar diseases, such as chronic infections, systemic autoimmune diseases, medication or exposure, and certain malignancies [3].

IMN is considered an antibody-mediated kidney disease where IgG autoantibodies from subepithelial immune complexes with autoantigens are expressed on the podocyte cell surface [4]. Sublethal damage to the overlying podocyte results in cellular simplification, as well as the disruption of the glomerular filtration barrier, leading to proteinuria along with other nephrotic syndrome manifestations [5].

Presently, renal biopsy constitutes the gold standard for the IMN diagnosis. Nevertheless, a repeat renal biopsy is not effective for evaluating disease severity, as well as progression considering it is an invasive method. Recently, many novel plasma or urine biosignatures have been developed, among which aPLA2R is the most commonly used because it is highly specific [6]. However, it is not very sensitive (between 52% and 78%) [7]. Thus, it is essential to explore novel noninvasive diagnostic biomarkers.

Exosomes are lipid bilayer membrane-originated vesicles from endocytic compartments that are 30-120 nm and occur in nearly all biofluids, such as urine [8–10]. Because exosomes possess cell-type-distinct signatures, they have been suggested as predictive biosignatures for a variety of clinical conditions [11, 12]. Urinary exosomes consist of proteins, mRNAs, and miRNAs which are produced by glomerular (podocytes, endothelial, and mesangial cells) and tubular cells. Thus, urinary exosomes may provide sensitive and accurate biomarkers for renal dysfunction and structural injury [13]. Numerous studies have uncovered distinct urinary exosomal miRNA expression patterns in individuals with kidney disease [10, 14–16]. In IMN, several exosome-derived circular RNAs are significantly differentially expressed in exosomes from serum and urine [17]. Thus, comprehensive analysis of sRNAs (including miRNAs) from urinary exosomes of IMN patients could provide useful disease biomarkers.

Herein, we analysed differences in urinary exosomal sRNA (including miRNA) patterns between IMN patients and healthy controls (CON) and uncovered both specifically expressed and differentially expressed miRNAs. The findings could enhance the design of prospective molecular targets for IMN diagnosis.

## 2. Materials and Methods

### 2.1. Patients

We screened eleven people in this study, belonging to two groups: (1) an IMN group (six people) and (2) a CON group (five people). All IMN patients were identified based on aPLA2R tests and renal biopsies performed at the Zhejiang Provincial People's Hospital, Zhejiang's Department of Nephrology, China. The baseline demographic and clinical data were documented at the time of kidney biopsy. Five healthy volunteers from the Physical Examination Center were recruited in the study as controls. The Zhejiang Provincial People's Hospital's ethics committee approved this study, and all work was carried out as per the Zhejiang Provincial People's Hospital ethical standards. Informed consent was given by all subjects.

### 2.2. Sample Acquisition and Purification of Exosomes

Whole-stream early morning urine samples were collected from each patient, as well as healthy control. Upon collection, urine samples were transferred to centrifuge tubes and span for 10 min at 2000 × g at 4°C. Afterwards, we aliquoted the supernatant into to fresh centrifuge tubes and span for 30 min at 10,000 × g at 4°C, and then, filtration through a 0.45 *μ*m filter was performed. The collected filtered liquid was used for exosome purification using an exosome extraction kit (Wako Pure Chemical Industries, Osaka, Japan) as per the manufacturer's provided protocol. Briefly, the sample concentration to 1 mL was conducted with the Amicon Ultra-15 Ultracel-100 K device (Merck KGaA, Darmstadt, Germany). The concentrated sample was inoculated with Streptavidin Magnetic Beads (60 mg) and 350 *μ*L Exosome Capture Immobilizing Buffer, 1 *μ*g of biotinylated mouse Tim4-Fc, and 50 *μ*L Exosome Binding Enhancer, and then overnight incubation was conducted at 4°C. Afterwards, beads were rinsed thrice with washing buffer the next day, and the bound extracellular vesicles (EVs) were eluted with Exosome Elution Buffer.

### 2.3. Transmission Electron Microscopy (TEM)

TEM was performed to assess exosome morphology using a PLSW 201901VIPI500-6 instrument (100biotech, Peking, China). Firstly, 10 *μ*L of the sample was introduced to a copper grid; then incubation was conducted for 1 min. A filter paper was employed to absorb excess liquid. A 10 *μ*L volume of phosphotungstic acid was added dropwise to the grid, incubated for 1 min, and excess liquid was again removed using a filter paper. After air drying, exosomes were visualised using an FEI Tecnai Spirit TEM T12 instrument (FEI, Hillsboro, OR, USA), and an electron-sensitive Olympus KeenView CCD camera was employed to acquire images.

### 2.4. Western Blotting

Following the manufacturer's protocols, total exosomal proteins were isolated by a Protein Extraction Kit (Applygen Technologies Inc., Beijing, China); then a BCA Protein Assay Kit was employed to assay protein concentration. Thereafter, fractionation of the proteins was conducted with an 8-10% SDS-PAGE, then transfer-embedded onto a polyvinylidene difluoride membrane. Subsequently, 5% nonfat milk was employed to block the membranes, which were then incubated with the primary antibodies: CD9 (1 : 1000, Bioss, Inc., Woburn, MA, USA), CD63 (1 : 1000, GeneTex, Irvine, CA, USA), and CD81 (1 : 800, GeneTex, Irvine, CA, USA) at a 1 : 1000 dilution, then with specified HRP-conjugated secondary antibodies. Signals were detected using chemiluminescence reagents (Beyotime, Shanghai, China).

### 2.5. RNA Extraction

The total Exosome RNA and Protein Isolation kit (Invitrogen, Life Technologies, USA) was employed to isolate the total RNA from the exosomes and maintained at -80°C for later use. Moreover, the Agilent 2200 TapeStation (Agilent Technologies, Santa Clara, CA, USA) was employed to assay the RNA quality for sequencing.

### 2.6. Sequencing of Small RNA and Data Analyses

High-throughput sequencing of urinary exosomes was performed for the six individuals with IMN and the five healthy controls, and sRNA libraries were processed using a NEBNext Multiplex Small RNA Library Prep Set for Illumina (NEB, Ipswich, MA, USA) as per the protocol provided by the manufacturer. In brief, we ligated the NEB 3′ SR Adaptor to the 3′-end of miRNAs, PIWI-interacting RNAs (piRNAs), and small interfering RNAs (siRNAs), and products were hybridized with the SR RT primer. The single-stranded DNA adaptor was then converted to a double-stranded DNA, and the 5′-end adapter was ligated to the 5′-ends of miRNAs. Besides, the first-strand cDNA was synthesised with the M-MuLV Reverse Transcriptase, followed by PCR amplification by the LongAmp Taq 2x Master Mix with SR Primer for Illumina and an index primer. Afterwards, we purified the PCR products by polyacrylamide gel electrophoresis, and DNA fragments (140 to 160 bp sizes) were recovered and solubilised in 8 *μ*L of elution buffer. Thereafter, the library quality was assessed using an Agilent Bioanalyzer 2100 system.

RNA libraries were put through 50 bp single-end read sequencing on an Illumina HiSeq 2500 platform. Thereafter, we removed the adaptors, as well as the low-quality sequences from the raw sequencing data, and clean reads were obtained and used for successive assessment. Reads were mapped to the hg38 human reference genome [18], and miRBase 20.0 was used to uncover the miRNAs. sRNAs originated from repetitive genomic regions were identified using the RepeatMasker web resource [19]. The original sequencing data was included within the supplementary information file (available here). The DEGseq (2010) R package was employed to perform differential expression analysis [20]. Differentially expressed miRNAs were those that satisfied the criteria of fold change ≥ 2 and *p* < 0.05.

### 2.7. Prediction of Target Genes and Functional Annotation

Target genes of exosomal miRNAs were predicted using TargetScan (http://www.targetscan.org) and Funrich software 3.1.3. Gene Ontology (GO) enrichment assessment, and Kyoto Encyclopedia of Genes and Genomes (KEGG) pathway assessment was conducted based on the DAVID online web resource (https://david.ncifcrf.gov/).

## 3. Results

### 3.1. Patient Characteristics

The demographic and the baseline clinical information of the participants is indicated in Table 1. The 24-hour proteinuria was remarkably higher in the IMN group in contrast with the controls, and the serum albumin was remarkably lower in the IMN group (all *p* < 0.001).

### 3.2. Differentially Expressed miRNA Patterns of Urinary Exosomes from the IMN and CON Groups

**Figure 1:**
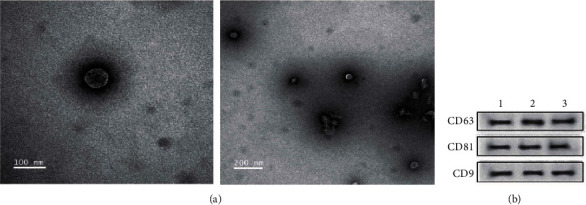
Authentication of urinary exosomes. (a) Transmission electron microscopy (TEM) images indicating exosome morphology. (b) Levels of CD81, CD9, and CD63 proteins measured by western blotting.

**Figure 2:**
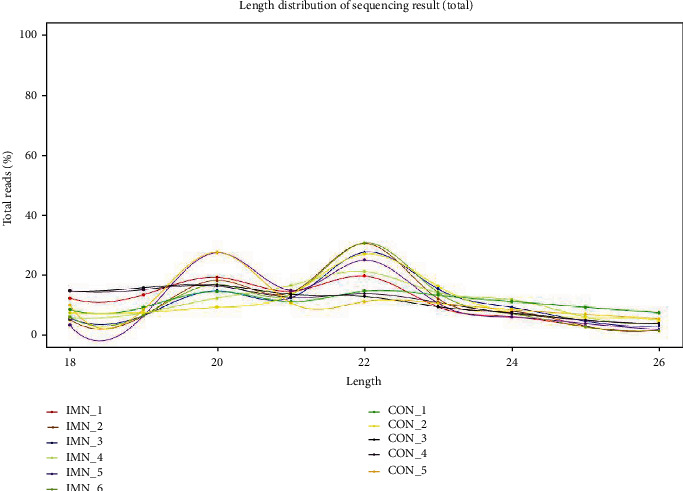
Analysis of small RNAs (sRNAs) contained in exosomes. Most of the sRNAs in exosomes are 20 nt and 22 nt in length, consistent with previous reports.

To assess the expression patterns of miRNAs in urine exosomes originated from healthy controls and IMN individuals, exosomes from each urine sample were extracted as described above, visualised by TEM, and the urine exosomes appeared to be circular (Figure 1(a)). Western blotting verified that the exosomal biomarkers CD9, CD63, and CD81 were present (Figure 1(b)). Total RNA was isolated from exosomes and assayed by an Agilent 2200 Bioanalyzer to provide size profiles and measure the concentration. The results revealed that sRNAs, and especially miRNAs, were abundant in exosomes (Figure 2).

**Figure 3:**
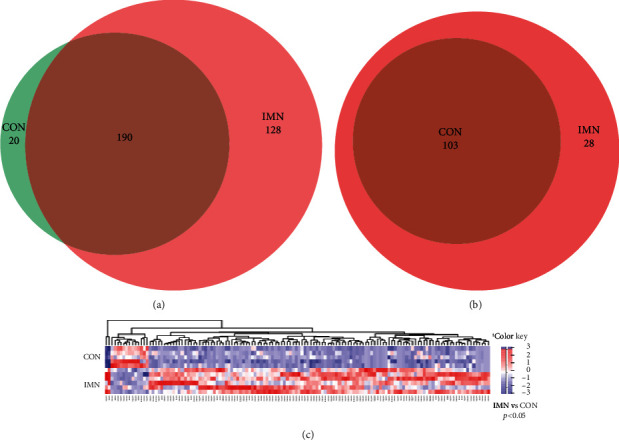
Expression profiling of miRNAs in urinary exosomes originated from the CON and IMN groups. (a) Venn diagram illustrating overlapping miRNAs in the two groups. (b) Venn diagram indicating overlapping differentially expressed miRNAs among the two groups (fold change ≥ 2 and *p* < 0.05). (c) Heatmap illustrating the expression of differentially expressed miRNAs in the two groups (fold change ≥ 2 and *p* < 0.05). The colour key designates the expression levels of miRNAs ranging from low (blue) to high (red).

We then performed high-throughput sequencing of miRNAs in exosomes in urine samples of individuals with IMN, as well as healthy controls, and 210 and 318 miRNAs were identified in the CON and IMN groups, respectively, by miRBase20.0/miRBase1 (Figure 3(a)). Furthermore, 131 miRNAs were remarkably differentially expressed (fold change ≥ 2 and *p* < 0.05; Figures 3(b) and 3(c)).

### 3.3. Specifically Expressed and Differentially Expressed miRNAs in the IMN and CON Groups

**Figure 4:**
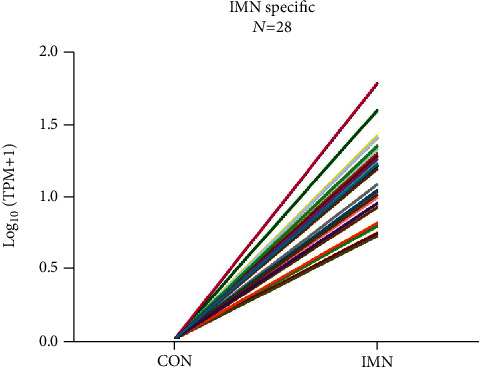
Specifically expressed miRNAs in urinary exosomes in the IMN group.

**Figure 5:**
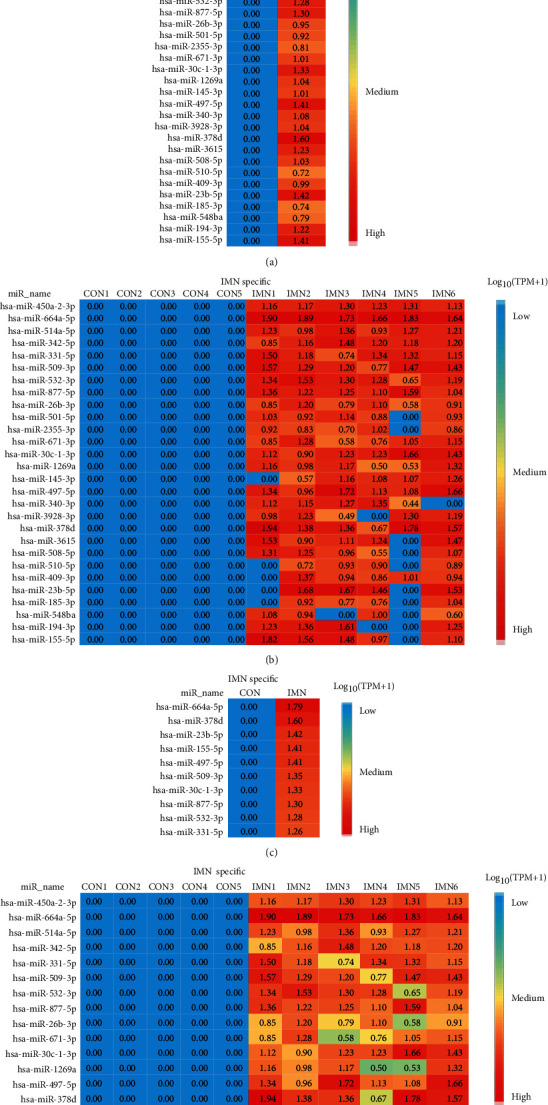
Specifically expressed miRNAs in urinary exosomes originated from the CON and IMN groups. (a) Heatmap illustrating 28 specifically expressed miRNAs in the CON and IMN groups. (b) Heatmap illustrating 28 specifically expressed miRNAs in individuals. (c) Heatmap illustrating the top 10 specifically expressed miRNAs in the CON and IMN groups. (d) Heatmap illustrating specifically expressed miRNAs expressed in all IMN individuals. The colour key indicates the expression levels of specifically expressed miRNAs ranging from low (blue) to high (red). The log_10_ (TPM) values are indicated.

**Figure 6:**
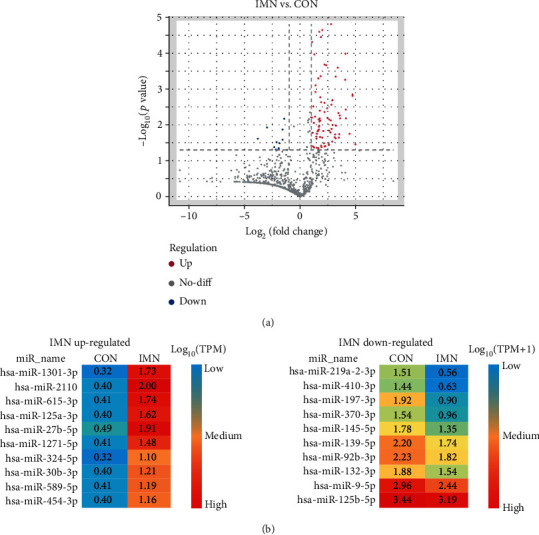
Differential expression of exosome miRNAs in the CON and IMN groups. (a) Volcano plot illustrating differentially expressed miRNAs in the CON and IMN groups. Red dots denote upregulated miRNAs, and blue dots denote downregulated miRNAs (fold change ≥ 2 and *p* < 0.05). (b) Heatmap illustrating the top 10 upregulated as well as downregulated coexpressed differential miRNAs. The colour key indicates the expression levels of specifically expressed miRNAs ranging from low (blue) to high (red). The log_10_ (TPM) values are indicated.

Among the 131 differentially expressed miRNAs, we uncovered 28 specifically expressed miRNAs between the CON and IMN groups (Figures 4 and 5(a)). Additionally, we found that several specifically expressed miRNAs were not expressed in some individuals in the IMN group (Figure 5(b)). Thus, we explored the top 10 distinctly expressed miRNAs in the two groups (Figure 5(c)) and the specifically expressed miRNAs expressed in all IMN individuals (Figure 5(d)). We also identified 108 differentially coexpressed miRNAs in the IMN and CON groups, of which 95 were upregulated and 13 were downregulated as indicated in Figure 6(a). The top 10 upregulated and downregulated coexpressed differential miRNAs are shown in Figure 6(b).

### 3.4. Prediction of Target Genes and GO/Pathway Assessment

**Figure 7:**
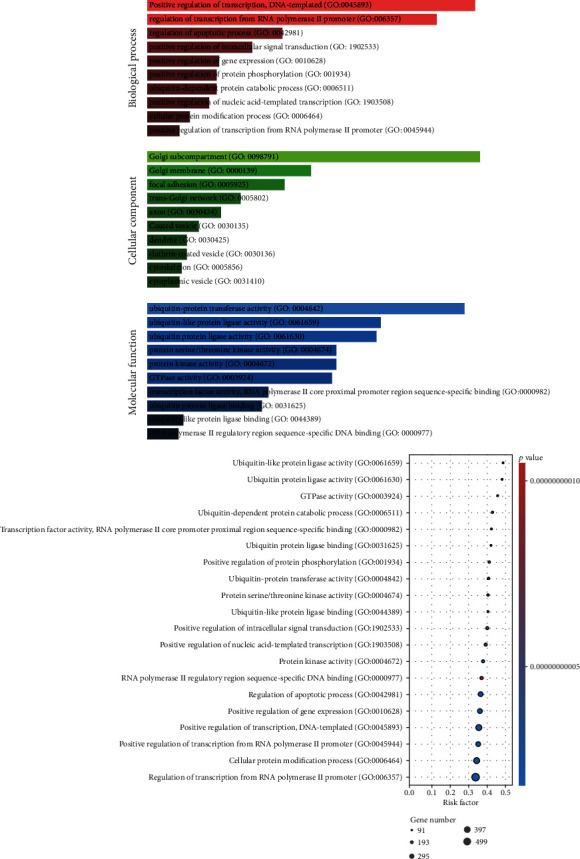
GO enrichment of the target genes. Colour intensity and the size of the nodes indicate the mean *p* value and number of genes, respectively.

**Figure 8:**
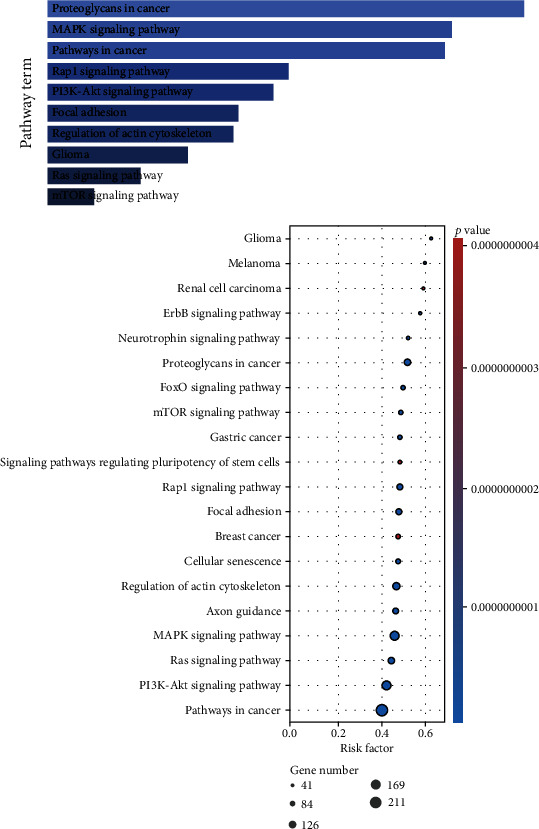
KEGG pathways of the target genes. The intensity of the colour and the size of the nodes indicate the mean *p* value and number of genes, respectively.

To confirm the differentially expressed miRNAs target genes, we firstly predicted the target on the basis of two algorithms, the FunRich3.1.3 and TargetScan, respectively. There were 4793 miRNA-target pairs collectively predicted by 2 algorithms. Next, the DAVID online database was employed to explore the GO as well as KEGG analyses of these target genes. The main biological process terms were related to positive modulation of transcription, modulation of transcription from RNA polymerase II promoter, and modulation of apoptotic process. The main cell component terms were associated with Golgi subcompartment, Golgi membrane, and focal adhesion. The main molecular function terms were linked to ubiquitin protein ligase activity, ubiquitin-protein transferase activity, and ubiquitin-like protein ligase activity (Figure 7). KEGG pathway analysis showed that proteoglycans in cancer, MAPK signalling cascades, and pathways in cancer were associated with target genes (Figure 8).

### 3.5. Other Kinds of Small RNAs in the IMN and CON Groups

**Figure 9:**
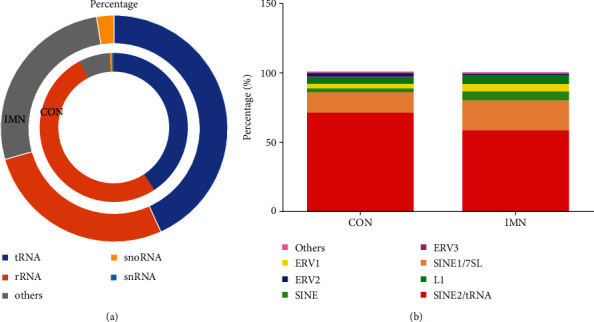
Classification and expression of sRNAs derived from the two groups. (a) Circles from the innermost to the outermost denote diverse kinds of small RNAs (indicated as different colours) from the two groups. (b) Expression levels of sRNAs from diverse repeated sequences among the two groups.

In addition to miRNAs, we also uncovered numerous other kinds of sRNAs in the two groups. Among them, sRNAs originated from transfer RNA (tRNA), ribosomal RNA (rRNA), and other kinds of RNA in exosomes accounting for the most significant proportion (Figure 9(a)). We also analysed repetitive regions of the genome and found that SINE2/tRNA was the most highly expressed, followed by SINE1/7SL, then SINE (Figure 9(b)).

## 4. Discussion

Over the past few decades, IMN incidence has increased worldwide, from 8.89% of primary glomerular disease in 2005 to 2009 to 19.11% in 2010 to 2014 [21]. Urinary exosomes contain large amounts of miRNAs, making urine a potentially useful biological sample for biomarkers related to renal dysfunction and structural injury [9, 10, 22]. Herein, we identified several differential miRNAs in urinary exosomes derived from the CON and IMN groups that are potentially informative biomarkers for IMN.

We identified 28 exosomal miRNAs that were specifically expressed in IMN (Figures 5(a) and 5(b)) and focused on the top 10 specifically expressed miRNAs, as well as those specifically expressed in all IMN individuals. Among these miRNAs, some have been reported previously to be closely linked to nephropathy. miR-378 suppresses apoptosis of podocytes through TRAF5 and thereby represses diabetic nephropathy (DN) progression, and it also regulates the protective function of mitogen-activated protein kinase 1 (MAPK1) in the stimulation of kidney cell fibrosis, as well as mesangial hypertrophy [23, 24]. miR-155-5p enhances oxalate- and calcium-triggered kidney oxidative stress injury by repressing matrix gla protein (MGP) expression and aggravating both inflammation and apoptosis in acute kidney injury tissues via the Jak2/Stat3 pathway [25]. miR-497 attenuates the endothelial-mesenchymal transition of glomerular endothelial cells via the modulation of rho linked coiled-coil containing protein kinase (ROCK) in diabetic nephropathy (DN) [26]. miR-532-3p is differentially expressed in membranous glomerulonephropathy (MGN) and chronic kidney disease (CKD) based on the analysis of renal biopsy sections [27, 28]. Additionally, multiple reports have opined that miR-23b has a close correlation with inflammation, as well as autoimmune diseases because it can enhance the oxLDL-triggered inflammatory response of macrophages via the A20/NF-*κ*B signalling cascade [29, 30].

Overall, 108 miRNAs were differentially coexpressed between the two groups. The top 10 up- and downregulated coexpressed miRNAs are shown in Figure 6(b), and seven of the miRNAs are known to be associated with renal diseases. miR-9-5p confers a protective response to chronic kidney injury, as well as renal fibrosis [31]. miR-92b-3p mediates advanced glycation end product- (AGE-) triggered development of renal abnormalities in rats with DN [32]. miR-125b-5p might be prospective biosignature for obstructive renal injury in individuals with ureteral obstruction linked to renal function [33]. miR-132-3p is expressed across the kidney cortex in mice, as well as humans with severe kidney damage or fibrosis [34]. The miR-139-5p expression level in kidney tissues of IRI-treated mice is reduced to 40.4% relative to healthy controls [35]. miR-145-5p may be a modulator of DN by inhibiting high glucose- (HG-) triggered apoptosis via targeting of Notch1, then dysregulating apoptotic factors [36].

Additionally, four miRNAs may have a potential impact on renal diseases. miR-27b may inhibit angiogenesis and fibroblast activation via the PI3K/AKT signalling pathway [37]. miR-615-3p enhances the phagocytic potential of splenic macrophages through targeting ligand-dependent nuclear receptor corepressor [38]. miR-197-3p is dominantly implicated in signalling cascades resulting in cytokine production [39, 40].

These specifically expressed exosomal miRNAs as well as differentially coexpressed exosomal miRNAs provide potential biomarkers for IMN.

To explore further about the functions of these differentially expressed miRNAs, further GO and KEGG assessments were conducted on the basis of those target genes of differentially expressed miRNAs. Remarkably, a lot of enriched GO terms were related to ubiquitin and apoptotic. Previous studies have proved that endoplasmic reticulum stress, autophagy, and ubiquitin-proteasome system serve a pivotal role in the onset of proteinuric kidney disease [41, 42]. For KEGG pathway assessment, we revealed a lot of target genes were enriched in the PI3K/AKT/mTOR cascade which was a well-known autophagy pathway. As in our previous study, we demonstrated that autophagy participates in the podocyte injury in IMN [43].

We also analysed repetitive regions of the genome in exosomes. Interestingly, the percentages of sRNAs from SINE1/7SL were slightly increased in the IMN group (Figure 2(b)). A previous study reported that cellular stress such as virus infection might cause upregulation of SINE elements [44]. However, no relation was found between IMN and SINE in the present work. The functional analysis of these repetitive regions of the genome in IMN individuals may be an exciting new area of research.

## 5. Conclusion

In conclusion, our findings demonstrate, for the first time, a remarkable difference in urinary exosomal miRNAs and repetitive region-derived sRNAs between individuals with IMN and healthy controls. The findings may promote the development of promising molecular targets for IMN.

## Figures and Tables

**Table 1:** The demographic, as well as the baseline clinical information of the study participants.

Group	IMN group	CON group	*p* value
Gender (female/male)	3/3	3/2	0.740
Age (years), median (range)	55.8 (26-72)	55.8 (26-64)	1.000
Urinary protein excretion (g/24 h)	5.89 (2.95-8.43)	/	/
Serum creatinine (*μ*mol/L)	75.03 (56.7-109.8)	75.64 (61.9-91.2)	0.953
Serum urea nitrogen (mmol/L)	5.19 (2.97-7.69)	4.59 (3.38-5.74)	0.503
eGFR (mL/min/1.73 m^2^)	98.96 (63.79-133.47)	97.9 (88.73-107.44)	0.926
APLA2R (U/mL)	145.38 (51.76-323.9)	/	/
Serum albumin (g/L)	21.95 (15.1-32.1)	43.68 (42.8-45.3)	0.006
Hypertension, *n* (%)	2 (33.33%)	2 (40%)	0.819
Diabetes, *n* (%)	0 (0.0%)	0 (0.0%)	/

## Data Availability

The data used to support the findings of this study are included within the supplementary information file(s).

## Supplementary Materials

Supplementary Materials Supplementary file 1 is the raw data used to support the findings of this study.
